# The effects of 8 weeks of whey or rice protein supplementation on body composition and exercise performance

**DOI:** 10.1186/1475-2891-12-86

**Published:** 2013-06-20

**Authors:** Jordan M Joy, Ryan P Lowery, Jacob M Wilson, Martin Purpura, Eduardo O De Souza, Stephanie MC Wilson, Douglas S Kalman, Joshua E Dudeck, Ralf Jäger

**Affiliations:** 1 Department of Health Sciences and Human Performance, The University of Tampa, Tampa, FL 33606, USA; 2 College of Professional Studies, North-eastern University, Boston, MA 02115, USA; 3 Increnovo LLC, 2138 E Lafayette Pl, Milwaukee, WI 53202, USA; 4 Laboratory of Neuromuscular Adaptations to Strength Training, School of Physical Education and Sport, University of São Paulo, São Paulo, Brazil; 5 Department of Nutrition, IMG Performance Institute, IMG Academies, Bradenton, FL, USA; 6 Department of Nutrition and Endocrinology, Miami Research Associates, Miami, FL, USA

**Keywords:** Protein Quality, Leucine, Whey, Rice

## Abstract

**Background:**

Consumption of moderate amounts of animal-derived protein has been shown to differently influence skeletal muscle hypertrophy during resistance training when compared with nitrogenous and isoenergetic amounts of plant-based protein administered in small to moderate doses. Therefore, the purpose of the study was to determine if the post-exercise consumption of rice protein isolate could increase recovery and elicit adequate changes in body composition compared to equally dosed whey protein isolate if given in large, isocaloric doses.

**Methods:**

24 college-aged, resistance trained males were recruited for this study. Subjects were randomly and equally divided into two groups, either consuming 48 g of rice or whey protein isolate (isocaloric and isonitrogenous) on training days. Subjects trained 3 days per week for 8 weeks as a part of a daily undulating periodized resistance-training program. The rice and whey protein supplements were consumed immediately following exercise. Ratings of perceived recovery, soreness, and readiness to train were recorded prior to and following the first training session. Ultrasonography determined muscle thickness, dual emission x-ray absorptiometry determined body composition, and bench press and leg press for upper and lower body strength were recorded during weeks 0, 4, and 8. An ANOVA model was used to measure group, time, and group by time interactions. If any main effects were observed, a Tukey post-hoc was employed to locate where differences occurred.

**Results:**

No detectable differences were present in psychometric scores of perceived recovery, soreness, or readiness to train (p > 0.05). Significant time effects were observed in which lean body mass, muscle mass, strength and power all increased and fat mass decreased; however, no condition by time interactions were observed (p > 0.05).

**Conclusion:**

Both whey and rice protein isolate administration post resistance exercise improved indices of body composition and exercise performance; however, there were no differences between the two groups.

## Background

Recommended levels for an adequate dietary protein intake for an adult is 0.8 grams per kilogram of body weight, the average daily intake level that is sufficient to meet the nutrient requirement of nearly all healthy individuals. The protein requirements are based on nitrogen balance, trying to achieve a balance between nitrogen intake and excretion. Protein recommendations for endurance and strength trained athletes range from 1.2 to 2.0 g/kg bw/d, reflecting the athlete’s nutritional goal to increase lean body mass [1,2]. The athlete has a choice of different animal (e.g. whey, casein, egg, beef, fish) or plant protein (e.g. soy, rice, pea, hemp) sources, differing in numerous ways such as the presence of allergens (lactose, soy), cholesterol, saturated fats, digestion rate (fast, intermittent, slow absorption of amino acids), or the relative amount of individual amino acids. In contrast to dairy protein, plant protein sources are more often lower in one or more essential amino acids failing to match the requirements of a complete protein (Table 1).

**Table 1:** Essential amino acids profile of a complete protein in comparison to whey protein isolate and rice protein isolate used in this study (Eurofins Analytical Laboratories, Metairie, LA)

**Essential amino acid [mg/g of protein]**	**Complete protein**	**Whey protein isolate**	**Rice protein isolate**
Tryptophan	7	22	14
Threonine	27	76	35
Isoleucine	25	70	41
Leucine	55	115	80
Lysine	51	101	31
Methionine + Cystine	25	48	49
Phenylalanine + Tyrosine	47	64	101
Valine	32	64	58
Histidine	18	18	22

Long term, periodized resistance training (RT) results in increases in skeletal muscle size and, ultimately, force generating capacity [3,4]. Sports nutrition scientists have attempted to increase training induced gains through a number of protocols, which generally attempt to augment and/or speed skeletal muscle regeneration. One such intervention has been to increase the provision of the branched chain amino acids (BCAAs), leucine, isoleucine, and valine, which make up more than one third of muscle protein [5]. The BCAAs are unique among the essential amino acids (EAAs) for their roles in protein metabolism [6], neural function [7-9], and blood glucose and insulin regulation [10]. Moreover, Garlick and colleagues [11] have found that BCAAs were able to stimulate skeletal muscle protein synthesis (MPS) to the same degree as all 9 EAAs. Of the BCAAs, only leucine was able to independently stimulate MPS [11]. It is well known that vigorous exercise can induce a net negative protein balance in response to both endurance and resistance training [12]. Norton and Layman proposed that consumption of BCAAs, namely leucine, could turn individuals from a negative to a positive whole-body protein balance after intense exercise [6]. In support, the consumption of a protein or EAA complex that contains sufficient leucine has been shown to shift protein balance to a net positive state after intense exercise training [6,13]. These findings led Norton and Wilson [14] to suggest that optimal protein intake per meal should be based on the leucine content of the protein consumed.

Early research indicates that 2-3 g, or up to 0.05 g/kg bodyweight, of leucine are required to maximize MPS [14-16]. However once this threshold has been reached, a protein’s beneficial effects on MPS effectively plateaus. For example, consuming 40 grams of egg protein (4 grams of leucine) did not enhance MPS over 20 grams of egg protein (2 grams of leucine) [17].

Plant-based proteins contain approximately 6–8% leucine, and in low doses, they do not increase MPS compared to animal-based proteins, which contain approximately 8–11% leucine [18,19]. However, if leucine is added to a plant-based protein, MPS rates are not significantly different from animal-based proteins [20]. Moreover at lower doses of protein (10% of energy), animal sources stimulate MPS to a greater degree than plant sources. However, at higher doses (30% of energy), both plant and animal-based proteins have reached the amount of leucine needed to optimize MPS, resulting in no differences between the sources [21].

To date however, no research has compared higher doses of plant to animal based protein following a resistance training intervention. Large doses of rice protein isolate, an allergen-free plant protein, containing 8% leucine may be a suitable form of protein to support muscle hypertrophy in combination with RT. Based on the available data, we hypothesize that higher doses of rice protein (48 g) will be comparable to an equally high dose of whey protein in its effects on lean mass and strength when given following RT. Therefore, the purpose of this study was to investigate the effects of higher doses of rice protein compared to equally high doses of whey protein on skeletal muscle hypertrophy, lean body mass, strength and power when given following 8 weeks of periodized RT in those individuals with previous RT experience.

## Methods

### Experimental design

Our study consisted of a randomized, double blind protocol consisting of individuals given either 48 grams of rice or 48 grams of whey protein isolate following an acute resistance exercise bout (phase 1) and following each session during an 8 week periodized training protocol (Phase 2). Phase 1 of the study investigated the effects of protein sources on recovery 48 hours following a high volume, hypertrophy oriented resistance-training session. Phase two occurred for the remaining eight-week RT protocol, which consisted of training each muscle group twice per week using a non-linear periodized RT model. Direct ultrasound determined muscle mass, dual emissions x-ray absorptiometery (DXA) determined body composition, maximal strength, and power were assessed collectively at the end of weeks 0, 4, and 8.

### Subjects

Twenty-four healthy males (21.3 ± 1.9 years, 76.08 ± 5.6 kg, 177.8 ± 12.3 cm) participated in the study. As inclusion criteria, it was required that all subjects cease taking nutritional supplements for three months prior to the study, had participated in RT at least 3 times per week for the past six months, and had a minimum of 1 year of RT experience. Subjects were carefully matched by age, body mass, strength, and resistance training experience, then randomly placed into either the rice (n = 12) or the whey (n = 12) group. All procedures were approved by the University of Tampa’s Institutional Review Board.

#### Phase 1 resistance training protocol

All subjects participated in a high volume resistance training session consisting of 3 sets of leg press, bench press, and military press, pull‒ups, bent over rows, barbell curls and extensions. Immediately following the workout, subjects consumed 48 grams of RPI or WPI respectively. Immediately prior to the exercise session and 48 hours post exercise, soreness, perceived readiness to train, and perceived recovery scale (PRS) measurements were taken. Soreness was measured on a visual analogue scale ranging from 0–10. With zero representing no soreness in the muscles at all, and 10 representing the worst muscle soreness ever experienced. PRS consists of values between 0–10, with 0–2 being very poorly recovered with anticipated declines in performance, 4–6 being low to moderately recovered with expected similar performance, and 8–10 representing high perceived recovery with expected increases in performance. Perceived readiness indicates how ready the subject felt they were to train. In this scale a 10 is the most ready an individual could be to train, while a 0 indicates the subject feels they are not ready at all to train.

### Resistance training protocol

Our resistance training protocol was a modified combination from Kraemer et al. [22] and Monteiro et al. [3]. These researchers found that a non-linear resistance-training program yielded greater results than a traditional or non periodized program in athletes. The program was designed to train all major muscle groups using mostly compound movements for the upper and lower body. The programmed, non-linear training split was divided into hypertrophy days consisting of 8–12 RM loads for 3 sets, with 60–120 seconds rest and strength days consisting of 2 to 5 RM loads for 3 sets for all exercises except the leg press and bench press which received 5 total sets. Weights were progressively increased by 2–5% when the prescribed repetitions could be completed. All training sessions were closely monitored by the researchers to ensure effort and intensity were maximal each training session.

### Strength, power, body composition and skeletal muscle hypertrophy testing

Strength was assessed via 1-RM testing of the leg press and bench press. Each lift was deemed successful as described by International Powerlifting Federation rules [23]. Body composition (lean body mass, fat mass, and total mass) was determined on a Lunar Prodigy DXA apparatus (software version, enCORE 2008, Madison, Wisconsin, U.S.A.). Skeletal muscle hypertrophy was determined via changes in ultrasonography determined combined muscle thickness of the biceps brachii and vastus lateralis (VL) and vastus intermedius (VI) muscles (General Electric Medical Systems, Milwaukee, WI, USA).

Power was assessed during a maximal cycling ergometry test. During the cycling test, the volunteer was instructed to cycle against a predetermined resistance (7.5% of body weight) as fast as possible for 10 seconds [24]. The saddle height was adjusted to the individual’s height to produce a 5–10° knee flexion while the foot was in the low position of the central void. A standardized verbal stimulus was provided to the subjects. Power output was recorded in real time by a computer connected to the Monark standard cycle ergometer (Monark model 894e, Vansbro, Sweden) during the 10-second sprint test. Peak power (PP) was recorded using Monark Anaerobic test software (Monark Anaerobic Wingate Software, Version 1.0, Monark, Vansbro, Sweden). From completion of wingate tests performed over several days, interclass correlation coefficient for peak power was 0.96.

### Supplementation and diet control

Two weeks prior to and throughout the study, subjects were placed on a diet consisting of 25% protein, 50% carbohydrates, and 25% fat by a registered dietician who specialized in sport nutrition. Subjects met as a group with the dietitian, and they were given individual meal plans at the beginning of the study. Daily total of calories were determined by the Harris-Benedict equation and tracked by weekly logs to ensure compliance. The protein supplement was administered under supervision of a laboratory assistant following resistance training, and it consisted of either 48 g of whey protein isolate (Nutra Bio Whey Protein Isolate (Dutch Chocolate), Middlesex, NJ) or 48 g of rice protein isolate (Growing Naturals Rice Protein Isolate (Chocolate Power) made with Oryzatein^®^ rice protein, Axiom Foods, Oro Valley, AZ) dissolved in 500 ml of water. The amino acid profile of the study material was analyzed by an independent analytical laboratory (Eurofins Analytical Laboratories, Metairie, LA) and is displayed in Table 2. Both the whey protein supplement and rice protein supplement were isonitrogenous, isocaloric, and macronutrient ratio matched.

**Table 2:** Amino acid profile of the study materials

**Amino acid [mg/g of protein]**	**Whey protein isolate**	**Rice protein isolate**
Alanine	54	54
Arginine	23	77
Aspartic Acid	118	87
Cystine	25	21
Glutamic Acid	191	174
Glycine	19	43
Histidine	18	22
Isoleucine	70	41
Leucine	115	80
Lysine	101	31
Methionine	23	28
Phenylalanine	33	53
Proline	64	45
Serine	52	49
Threonine	76	35
Tryptophan	22	14
Tyrosine	31	47
Valine	64	58

All supplements were tested by HFL Sports Science prior to use to ensure no contamination with steroids or stimulants according to ISO 17025 accredited tests.

### Statistics

An ANOVA model was used to measure group, time, and group by time interactions for both phase 1 and 2. If any main effects were observed, a Tukey post-hoc was employed to locate where differences occurred. All statistics were run using Statistica software (Statsoft, 2011).

## Results

### Phase 1

No differences existed between groups at baseline for any measure. There were no differences in the total amounts of weight lifted by the RPI (12296.3 ± 2412.6 kg) or WPI (11831.6 ± 2611.3 kg) group during the resistance training session. There was a significant time effect (p <0.05) for soreness, which increased in both the RPI (0.3 ± 0.6 to 5.6 ± 2.2) and WPI (0.3 ± 0.5 to 6.0 ± 1.9) groups, with no differences between groups (no condition X time effect). There was a significant time effect (p <0.05) for PRS, which decreased in both the RPI (9.1 ± 1.5 to 5.45 ± 1.5) and WPI (8.7 ± 2.6 to 5.6 ± 1.4) groups, with no differences between groups (no condition X time effect). There were no significant time or condition x time effects for perceived readiness to train, indicating that the subject’s perceived readiness had recovered within 48 hours.

### Phase 2

**Figure 1:**
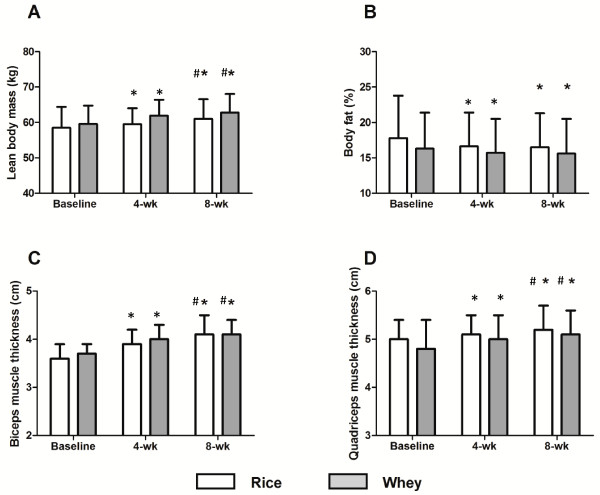
**Changes in (A) Lean Body mass, (B) Body fat, (C) Biceps muscle thickness and (D) Quadriceps muscle thickness.** * Indicates significantly different from baseline. # Indicates significantly different from week 4.

There was a significant time effect (p <0.01) for lean body mass, which increased in both the rice (58.5 ± 5.5 (baseline) to 59.5 ± 4.5 (week 4) to 61.0 ± 5.6 kg (week 8) and whey protein (59.6 ± 5.2 to 61.9 ± 4.5 to 62.8 ± 5.2 kg) conditions, with no differences between conditions (no condition X time effect). There was a significant time effect for body fat (p < 0.05), which decreased in both conditions, 17.8 ± 6.0 to 16.6 ± 4.8 to 15.6 ± 4.9 kg in the rice protein condition and 16.3 ± 5.1 to 15.7 ± 4.8 to 15.6 ± 4.9 kg in the whey protein condition, from pre to post training, with no differences between conditions (no condition X time effect). There was a significant time effect for quadriceps and biceps thickness (p < 0.05), which increased from pre to post training in the rice protein (5.0 ± 0.4 to 5.1 ± 0.4 to 5.2 ± 0.5 cm and 3.6 ± 0.3 to 3.9 ± 0.3 to 4.1 ± 0.4 cm, respectively) and whey protein (4.8 ± 0.7 to 5.0 ± 0.5 to 5.1 ± 0.5 cm and 3.6 ± 0.2 to 4.0 ± 0.3 to 4.1 ± 0.3 cm, respectively) conditions, with no differences between conditions (no condition X time effect). Body composition data is displayed in Figure 1.

There was a significant time effect (p < 0.01) for 1-RM bench press strength, which increased from baseline to week 8 in both the rice protein (85.9 ± 20.5 to 95.5 ± 21.4 kg) and whey protein (89.5 ± 18.5 to 98.5 ± 16.4 kg ) conditions, with no differences between groups (no condition X time effect). There was a significant time effect (p < 0.01) for 1-RM leg press strength, which increased from baseline to week 8 in both the rice (220.0 ± 38.5 to 286.8 ± 37.2 kg) and whey (209.5 ± 35.0 to 289.7 ± 40.1 kg) conditions, with no differences between conditions (no condition X time effect). There was a significant time effect for wingate peak power (p < 0.01), which increased from baseline to week 8 in both the rice protein (638.4 ± 117.2 to 753.9 ± 115.6 watts) and whey protein (687.1 ± 125.3 to 785.0 ± 101.1 watts) conditions, with no differences between conditions. Performance data is displayed in Table 3.

**Table 3:** Changes in strength and power

	**Baseline**	**Week 4**	**Week 8**
Bench Press (kg) Rice Protein Isolate	85.9 ± 20.5	91.6 ± 21.2	95.5 ± 21.4*
Bench Press (kg) Whey Protein Isolate	89.5 ± 18.5	95.5 ± 17.8	98.5 ± 16.4*
Leg Press (kg) Rice Protein Isolate	220.0 ± 38.5	266.4 ± 34.6	286.8 ± 37.2*
Leg Press (kg) Whey Protein Isolate	209.5 ± 35	259.5 ± 39.6	289.7 ± 40.1 *
Peak Power (W) Rice Protein Isolate	638.4 ± 117.2	692.5 ± 118.6	753.9 ± 115.6*
Peak Power (W) Whey Protein Isolate	687.1 ± 125	740.8 ± 115.4	785.0 ± 101.1*

Data is displayed as mean ± standard deviation, * denotes significant change from baseline (p ≤ 0.05).

## Discussion

The novel finding in the present study is that no significant condition by time interactions were observed between the rice protein and whey protein supplements on short term recovery or training-induced adaptations. Our findings support the proposed hypothesis that higher doses of rice protein (48 g) will be comparable to an equally high dose of whey protein in its effects on body composition and exercise performance after periodized RT. In other words, RPI supports changes in strength and body composition similarly to WPI.

Subjects were given either 48 g of protein in the form of a rice or whey protein supplement. At these doses, the rice protein supplement contained approximately 3.8 g of leucine whereas the whey protein supplement contained 5.5 g of leucine. At these doses, both supplements are predicted to reach levels necessary to optimize muscle protein accretion [14] they are also greater than the amounts observed in prior research [25,26]. Moore et al. [25] conducted a dose response study of an egg protein supplement comparing 0 g, 5 g, 10 g, 20 g, and 40 g of egg protein delivered after a bout of exercise. After consumption of the supplement, MPS rates were monitored for four hours. Their results suggested that MPS was maximally stimulated with 20 g of egg protein, which contains 1.7 g of leucine. It was also observed that at double that dose (40 g, 3.4 g of leucine), no significant differences in MPS occurred.

Chronic free leucine supplementation alone did not improve lean body or muscle mass during resistance training in the elderly, whereas it was able to limit the weight loss induced by malnutrition. Leucine-rich amino acid mixtures or proteins appeared more efficient than leucine alone to improve muscle mass and performance, thereby suggesting the efficacy of leucine depends on the presence of other amino acids. Small differences in protein digestion rates, differences in branched-chain amino acid content can impact the ability of the protein to maximize post exercise MPS. Available data on soy protein suggests that plant proteins might differ in their ability to support muscle protein accretion after resistance exercise [17,26]. For example, post exercise consumption of fat-free milk promotes greater hypertrophy during the early stages of resistance training in novice weightlifters when compared with isonitrogenous and isoenergetic fat-free soy protein [26]. Hartman et al. [26] conducted research comparing milk protein, to soy protein, to a maltodextrin control in untrained individuals. In Hartman’s study, 17.5 g of protein in the form of milk or soymilk was given immediately and one hour following exercise, while the control group received an isocaloric maltodextrin beverage. 17.5 g of protein from milk contains approximately 1.7 g of leucine, and 17.5 g of protein from soymilk would contain 1.4 g of leucine. Following a twelve week RT program, the milk protein group experienced greater increases in type II muscle fiber area. This study suggests that a moderate dose of milk protein increases lean mass to a greater extent than soy or a maltodextrin control when given following exercise. Soy proteins appear to support greater splanchnic rather than peripheral (i.e., muscle) protein synthesis and are converted to urea to a greater extent than are milk proteins. Alternatively, observed differences might be explained by differences in leucine content or absorption kinetics.

**Figure 2:**
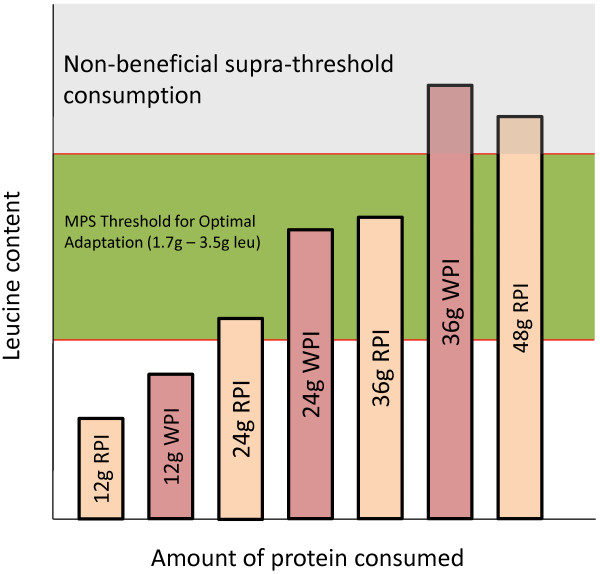
Theoretical model for protein dose and the anabolic response.

In the present study, the combined muscle thickness of the VI and VL increased in both the rice protein (0.2 cm) and whey protein (0.5 cm) conditions. Lean body mass increased in the rice protein condition by 2.5 kg, and it also increased in the whey protein condition by 3.2 kg. Combined bench press and leg press 1-RM strength increased in the rice protein condition by 76.4 kg and in the whey protein condition by 89.5 kg. However, no significant differences were observed between the two conditions for any measure. The collective findings of our study and others suggests that as the amount of protein consumed increases, the importance of the relative leucine content of the protein diminishes (see Figure 2) [21,27].

### Study limitations

Limitations of this study include the duration of the research and the lack of a non-supplemented control group. While no significant effects were observed between groups, potential differences in effects on body composition and exercise performance between groups may be more evident if examined over a longer duration. Without a non-supplemented control group, we cannot conclude how beneficial protein supplementation was to resistance training in this study.

## Conclusion

The present results suggest that differences in protein composition are of less relevance when protein is consumed in high doses throughout periodized RT. Rice protein isolate consumption post resistance exercise decreases fat-mass and increases lean body mass, skeletal muscle hypertrophy, power and strength comparable to whey protein isolate.

## Competing interests

The authors declare that they have no competing interests.

## Authors’ contribution

JMJ, RPL and JMW developed the study design, supervised and trained subjects, performed the statistical analysis, participated in data acquisition and drafting the manuscript. RJ developed the study design and participated in drafting the manuscript. MP assisted with the design of the study and drafting the manuscript. EODS assisted in drafting of the manuscript and performing the statistical analysis. SMCW was responsible for diet control and also assisted in drafting the manuscript. JED supervised subjects and assisted in drafting the manuscript. DSK assisted in study conception and writing of the manuscript. All authors read and approved the final manuscript.

## References

[B1] Phillips SM Dietary protein requirements and adaptive advantages in athletes Br J Nutr 2012 108 Suppl 2 S158 S167 23107527 10.1017/S0007114512002516

[B2] Campbell B Kreider RB Ziegenfuss T La Bounty P Roberts M Burke D Landis J Lopez H Antonio J International society of sports nutrition position stand: protein and exercise J Int Soc Sports Nutr 2007 4 8 10.1186/1550-2783-4-8 17908291 PMC2117006

[B3] Monteiro AG Aoki MS Evangelista AL Alveno DA Monteiro GA Picarro Ida C Ugrinowitsch C Nonlinear periodization maximizes strength gains in split resistance training routines Journal of strength and conditioning research / National Strength & Conditioning Association 2009 23 1321 1326 10.1519/JSC.0b013e3181a00f96 19528843

[B4] Turner A The science and practice of periodization: a brief review Strength and Conditioning Journal 2011 33 34 46

[B5] Kraemer WJ Ratamess NA Volek JS Hakkinen K Rubin MR French DN Gomez AL McGuigan MR Scheett TP Newton RU The effects of amino acid supplementation on hormonal responses to resistance training overreaching Metabolism 2006 55 282 291 10.1016/j.metabol.2005.08.023 16483870

[B6] Norton LE Layman DK Leucine regulates translation initiation of protein synthesis in skeletal muscle after exercise J Nutr 2006 136 533S 537S 16424142 10.1093/jn/136.2.533S

[B7] Blomstrand E A role for branched-chain amino acids in reducing central fatigue J Nutr 2006 136 544S 547S 16424144 10.1093/jn/136.2.544S

[B8] Newsholme EA Blomstrand E Branched-chain amino acids and central fatigue J Nutr 2006 136 274S 276S 16365097 10.1093/jn/136.1.274S

[B9] Davis JM Carbohydrates, branched-chain amino acids, and endurance: the central fatigue hypothesis Int J Sport Nutr 1995 5 Suppl S29 S38 7550256 10.1123/ijsn.5.s1.s29

[B10] Brosnan JT Brosnan ME Branched-chain amino acids: enzyme and substrate regulation J Nutr 2006 136 207S 211S 16365084 10.1093/jn/136.1.207S

[B11] Garlick PJ The role of leucine in the regulation of protein metabolism J Nutr 2005 135 1553S 1556S 15930468 10.1093/jn/135.6.1553S

[B12] Layman DK Role of leucine in protein metabolism during exercise and recovery Can J Appl Physiol 2002 27 646 663 10.1139/h02-038 12501002

[B13] Biolo G Tipton KD Klein S Wolfe RR An abundant supply of amino acids enhances the metabolic effect of exercise on muscle protein Am J Physiol 1997 273 E122 E129 9252488 10.1152/ajpendo.1997.273.1.E122

[B14] Norton L Wilson GJ Optimal protein intake to maximize muscle protein synthesis AgroFood industry hi-tech 2009 20 54 57

[B15] Paddon-Jones D Sheffield-Moore M Zhang XJ Volpi E Wolf SE Aarsland A Ferrando AA Wolfe RR Amino acid ingestion improves muscle protein synthesis in the young and elderly Am J Physiol Endocrinol Metab 2004 286 E321 E328 14583440 10.1152/ajpendo.00368.2003

[B16] Tipton KD Ferrando AA Phillips SM Doyle D Jr Wolfe RR Postexercise net protein synthesis in human muscle from orally administered amino acids Am J Physiol 1999 276 E628 E634 10198297 10.1152/ajpendo.1999.276.4.E628

[B17] Tang JE Moore DR Kujbida GW Tarnopolsky MA Phillips SM Ingestion of whey hydrolysate, casein, or soy protein isolate: effects on mixed muscle protein synthesis at rest and following resistance exercise in young men J Appl Physiol 2009 107 987 992 10.1152/japplphysiol.00076.2009 19589961

[B18] Wilkinson SB Tarnopolsky MA Macdonald MJ Macdonald JR Armstrong D Phillips SM Consumption of fluid skim milk promotes greater muscle protein accretion after resistance exercise than does consumption of an isonitrogenous and isoenergetic soy-protein beverage Am J Clin Nutr 2007 85 1031 1040 17413102 10.1093/ajcn/85.4.1031

[B19] Rasmussen BB Phillips SM Contractile and nutritional regulation of human muscle growth Exerc Sport Sci Rev 2003 31 127 131 10.1097/00003677-200307000-00005 12882478

[B20] Norton LE Wilson GJ Layman DK Moulton CJ Garlick PJ Leucine content of dietary proteins is a determinant of postprandial skeletal muscle protein synthesis in adult rats Nutr Metab 2012 9 67 10.1186/1743-7075-9-67 PMC3488566 22818257

[B21] Norton LE Wilson GJ Rupassar I Garlick PJ Layman DK Leucine contents of isonitrogenous protein sources predict post prandial skeletal muscle protein synthesis in rats fed a complete meal FASEB 2009 22 227.224

[B22] Kraemer WJ Hatfield DL Volek JS Fragala MS Vingren JL Anderson JM Spiering BA Thomas GA Ho JY Quann EE Effects of amino acids supplement on physiological adaptations to resistance training Medicine and science in sports and exercise 2009 41 1111 1121 10.1249/MSS.0b013e318194cc75 19346975

[B23] Gilbert G Lees A Changes in the force development characteristics of muscle following repeated maximum force and power exercise Ergonomics 2005 48 1576 1584 10.1080/00140130500101163 16338723

[B24] Smith JC Fry AC Weiss LW Li Y Kinzey SJ The effects of high-intensity exercise on a 10-second sprint cycle test Journal of strength and conditioning research / National Strength & Conditioning Association 2001 15 344 348 11710663

[B25] Moore DR Robinson MJ Fry JL Tang JE Glover EI Wilkinson SB Prior T Tarnopolsky MA Phillips SM Ingested protein dose response of muscle and albumin protein synthesis after resistance exercise in young men Am J Clin Nutr 2009 89 161 168 19056590 10.3945/ajcn.2008.26401

[B26] Hartman JW Tang JE Wilkinson SB Tarnopolsky MA Lawrence RL Fullerton AV Phillips SM Consumption of fat-free fluid milk after resistance exercise promotes greater lean mass accretion than does consumption of soy or carbohydrate in young, novice, male weightlifters Am J Clin Nutr 2007 86 373 381 17684208 10.1093/ajcn/86.2.373

[B27] Pasiakos SM McClung HL McClung JP Margolis LM Andersen NE Cloutier GJ Pikosky MA Rood JC Fielding RA Young AJ Leucine-enriched essential amino acid supplementation during moderate steady state exercise enhances postexercise muscle protein synthesis Am J Clin Nutr 2011 94 809 818 10.3945/ajcn.111.017061 21775557

